# Single-cell transcriptomes and *runx2b^−/−^* mutants reveal the genetic signatures of intermuscular bone formation in zebrafish

**DOI:** 10.1093/nsr/nwac152

**Published:** 2022-08-02

**Authors:** Chun-Hong Nie, Shi-Ming Wan, Yu-Long Chen, Ann Huysseune, Ya-Ming Wu, Jia-Jia Zhou, Alexandre Wagner Silva Hilsdorf, Wei-Min Wang, Paul Eckhard Witten, Qiang Lin, Ze-Xia Gao

**Affiliations:** College of Fisheries, Key Lab of Freshwater Animal Breeding, Ministry of Agriculture and Rural Affairs/Key Lab of Agricultural Animal Genetics, Breeding and Reproduction of Ministry of Education/Engineering Research Center of Green Development for Conventional Aquatic Biological Industry in the Yangtze River Economic Belt, Ministry of Education, Huazhong Agricultural University, Wuhan 430070, China; Hubei Hongshan Laboratory, Wuhan 430070, China; College of Fisheries, Key Lab of Freshwater Animal Breeding, Ministry of Agriculture and Rural Affairs/Key Lab of Agricultural Animal Genetics, Breeding and Reproduction of Ministry of Education/Engineering Research Center of Green Development for Conventional Aquatic Biological Industry in the Yangtze River Economic Belt, Ministry of Education, Huazhong Agricultural University, Wuhan 430070, China; Hubei Hongshan Laboratory, Wuhan 430070, China; CAS Key Laboratory of Tropical Marine Bio-Resources and Ecology, South China Sea Institute of Oceanology, Innovation Academy of South China Sea Ecology and Environmental Engineering, Chinese Academy of Sciences, Guangzhou 510301, China; College of Fisheries, Key Lab of Freshwater Animal Breeding, Ministry of Agriculture and Rural Affairs/Key Lab of Agricultural Animal Genetics, Breeding and Reproduction of Ministry of Education/Engineering Research Center of Green Development for Conventional Aquatic Biological Industry in the Yangtze River Economic Belt, Ministry of Education, Huazhong Agricultural University, Wuhan 430070, China; Hubei Hongshan Laboratory, Wuhan 430070, China; Department of Biology, Ghent University, Ghent B-9000, Belgium; College of Fisheries, Key Lab of Freshwater Animal Breeding, Ministry of Agriculture and Rural Affairs/Key Lab of Agricultural Animal Genetics, Breeding and Reproduction of Ministry of Education/Engineering Research Center of Green Development for Conventional Aquatic Biological Industry in the Yangtze River Economic Belt, Ministry of Education, Huazhong Agricultural University, Wuhan 430070, China; Hubei Hongshan Laboratory, Wuhan 430070, China; College of Fisheries, Key Lab of Freshwater Animal Breeding, Ministry of Agriculture and Rural Affairs/Key Lab of Agricultural Animal Genetics, Breeding and Reproduction of Ministry of Education/Engineering Research Center of Green Development for Conventional Aquatic Biological Industry in the Yangtze River Economic Belt, Ministry of Education, Huazhong Agricultural University, Wuhan 430070, China; Hubei Hongshan Laboratory, Wuhan 430070, China; Unit of Biotechnology, University of Mogi das Cruzes, Mogi das Cruzes, Săo Paulo 08780-911, Brazil; College of Fisheries, Key Lab of Freshwater Animal Breeding, Ministry of Agriculture and Rural Affairs/Key Lab of Agricultural Animal Genetics, Breeding and Reproduction of Ministry of Education/Engineering Research Center of Green Development for Conventional Aquatic Biological Industry in the Yangtze River Economic Belt, Ministry of Education, Huazhong Agricultural University, Wuhan 430070, China; Hubei Hongshan Laboratory, Wuhan 430070, China; Department of Biology, Ghent University, Ghent B-9000, Belgium; CAS Key Laboratory of Tropical Marine Bio-Resources and Ecology, South China Sea Institute of Oceanology, Innovation Academy of South China Sea Ecology and Environmental Engineering, Chinese Academy of Sciences, Guangzhou 510301, China; College of Fisheries, Key Lab of Freshwater Animal Breeding, Ministry of Agriculture and Rural Affairs/Key Lab of Agricultural Animal Genetics, Breeding and Reproduction of Ministry of Education/Engineering Research Center of Green Development for Conventional Aquatic Biological Industry in the Yangtze River Economic Belt, Ministry of Education, Huazhong Agricultural University, Wuhan 430070, China; Hubei Hongshan Laboratory, Wuhan 430070, China; Guangdong Laboratory for Lingnan Modern Agriculture, Guangzhou 510000, China

**Keywords:** teleosts, zebrafish, intermuscular bones, scRNA-seq, tendon-osteoblast cell lineage, *runx2b*, genetic breeding

## Abstract

Intermuscular bones (IBs) are mineralized spicules, present in the myosepta of many, but not all, teleost species. IBs are often small and sharp, and they consequently limit how the fish can be processed; the IBs may cause injury or trauma if lodged in consumers’ throats or mouths, and therefore affect the appeal of the fish to many consumers. The development of IBs in teleosts is still not fully understood and the molecular basis of IB development remains to be established. Here, the characteristics of IB tissue are evaluated based on single-cell transcriptomics in wild-type zebrafish. The analysis defined 18 distinct cell types. Differentiation trajectories showed that IBs are derived from tendons and that a core tendon-osteoblast cell lineage is related to IB formation. In particular, the functions of 10 candidate genes were evaluated via CRISPR-Cas9 mutants. Among those, *runx2b^−/−^* mutants completely lost IBs, while swimming performance, growth and bone mineral density were not significantly different from *runx2b^+/+^* zebrafish. Comparative single-cell RNA sequencing (scRNA-seq) analysis in *runx2b^−/−^* and *runx2b^+/+^* zebrafish revealed the role of osteoblasts in IB formation. In addition, differentially expressed genes were enriched in the transforming growth factor β/bone morphogenetic protein (TGF-β/BMP) pathway after *runx2b* deletion. This study provides evidence for the crucial role of *runx2b* regulation in IB formation. Genetic breeding can target *runx2b* regulation and generate strains of commercial fish species without IBs, which can improve the safe consumption and economic value of many farmed fish species.

## INTRODUCTION

Fish products are part of the healthy diet of billions of people and represent an important source of animal protein for consumers worldwide. Today, to protect marine fish stocks, fish products must increasingly come from sustainable aquaculture [1–4]. From 1961 to 2018, the fraction of farmed finfish in per capita human consumption steadily increased (Fig. S1A and Table S1) [5] and the consumption of finfish has become indispensable for consumers worldwide. Yet an important drawback for human consumption is the presence of numerous intermuscular bones (IBs), located in the dorsal and caudal muscle tissue. Nearly half of the top 20 farmed fish species are carps or their relatives (Cyprinidae), which possess IBs (Fig. S1B and C). Removing the IBs from the musculature during processing has proven to be very difficult. Therefore, developing a novel strategy for aquaculture species by advanced breeding technology, to remove their IBs, would represent a major breakthrough, eradicating all risk of injury to consumers’ throats and/or digestive organs [6–8]. Species such as grass carp (*Ctenopharyngodon idellus*), common carp (*Cyprinus carpio*), rohu carp (*Labeo rohita*), tambaqui (*Colossoma macropomum*) and blunt snout bream (*Megalobrama amblycephala*) are candidates for such a strategy. Its feasibility was brought to light by the identification, in one breeding population of tambaqui, of some healthy individuals that lacked IBs, whereas individuals normally possess a significant number of IBs [9].

Generally, IBs are regarded as ossified myoseptal tendons. Amongst extant vertebrates, they occur only in teleosts [7,10]. Within teleosts, IBs evolved from being few in number in primitive taxa (e.g. Leptolepids) to being abundant in more advanced teleosts (e.g. Clupeiformes), and finally, to being scarce or not present at all in even more advanced, recently evolved teleosts (e.g. Acanthomorpha) [7,10]. Given that more advanced teleost species have lost IBs, uncertainties remain about the function of IBs. On the other hand, studies on the number, morphology and ossification processes of teleost fish IBs have revealed that their development occurs relatively independently from, and lags behind that of, other bone elements [7,10,11]. Biologists have also used a variety of techniques to explore the tissue origin and differentiation characteristics of IBs [8,12,13]. It was noted that the origin of IBs lies in the intramembranous ossification of the myoseptal tendon [7,10]. In mammals, the osteogenic differentiation capacity of tendon progenitor cells has been identified, providing novel insights into the origin of the cells involved in IB formation in teleosts [14]. Transcriptome analysis in *M. amblycephala* has revealed some candidate KEGG (Kyoto Encyclopedia of Genes and Genomes) pathways and key regulation genes for IB formation [12,13]. Considering that *Scx* promotes tendon stem cell development in mice, *scxa^–/–^* zebrafish were constructed. These displayed a lower number of IBs and the reduced differentiation ability of tendon progenitor cells, corroborating the view that IBs originate from tendon progenitor cells [8]. However, not only IBs, but also ribs showed developmental defects in *scxa^–/–^* zebrafish [8,15], indicating that the key regulation factors of IBs need to be further explored. Moreover, the differentiation pattern and molecular mechanisms underlying IB differentiation in teleosts, from tendon progenitor cells to osteoblasts, remain unclear.

To understand IB formation, we have turned to the zebrafish (*Danio rerio*), not only for its status as a model organism, but mostly because the zebrafish is a member of the carp family (Cyprinidae) and shares the IB type (epineurals and epipleurals) of farmed carp species. Here, we present a single-cell transcriptomic analysis of the intermuscular tissue (where the IBs develop) of 60 dpf wild-type zebrafish, i.e. in the phase of rapid growth and mineralization of IBs. The aims were to acquire characteristic features at the single-cell level and to elucidate the differentiation trajectory of cell clusters related to IB formation. Some putative key genes were screened based on the cell differentiation trajectory of IBs and tendons, followed by validation of their function through clustered regularly interspaced short palindromic repeats (CRISPR)/CRISPR-associated protein 9 (CRISPR-Cas9) technology. Our findings can provide important information for revealing the molecular mechanism behind IB formation, with major implications in the future for breeding farmed fish strains without IBs.

## RESULTS

### ScRNA-seq reveals the cellular composition of the region where IBs develop

**Figure 1.**
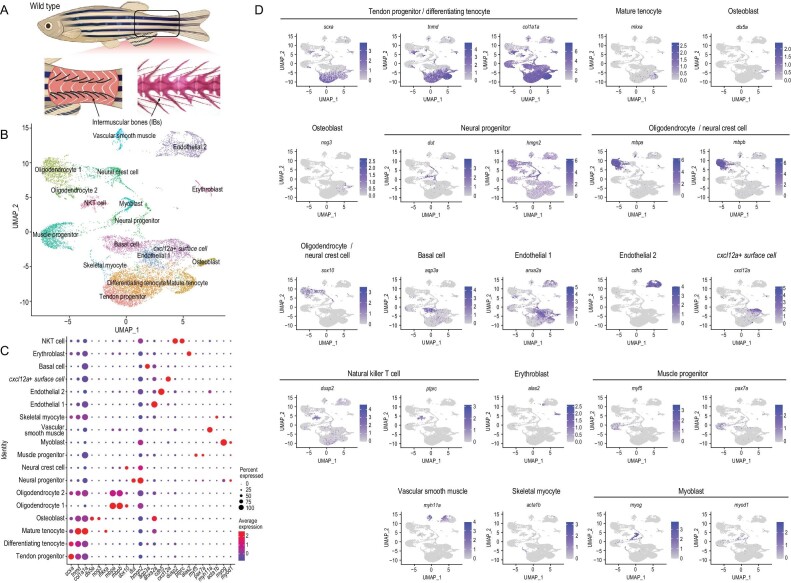
Global patterns of single-cell expression profiles of the tissue of origin of intermuscular bones (IBs) in wild-type zebrafish, and identification of cell types. (A) Pattern of tissue collection for scRNA-seq. (B) Uniform manifold approximation and projection (UMAP) visualization of 18 subsets from 13 075 cells from IB-containing muscle in wild-type zebrafish. (C) Dot plots showing the expression of curated feature genes in 18 subsets. Dot sizes represent the proportion of cells expressing a specific gene in the indicated subset and color bars represent the gene expression levels. (D) UMAP visualization of the expression of curated feature genes for tenocyte and osteoblast identification. The color bar represents the gene expression levels.

To analyze the cell diversity of the tissue where IBs develop, transcriptomes of 13 075 cells from the tail muscle of 60 dpf wild-type zebrafish (containing typical IB types—epineurals and epipleurals) were obtained through scRNA-seq (Fig. 1A). After the transcriptomes were pooled and clustered, 18 distinct cell clusters were generated and visualized using the uniform manifold approximation and projection (UMAP) approach (Fig. 1B). Next, cell identities were assigned based on marker genes that were significantly expressed in specific clusters (Fig. 1B, Fig. S2 and Table S2). Based on the expression pattern of specific marker genes (*dlx5a*, *nog3*, etc.), we defined osteoblasts as being closely related to IB formation (Fig. 1C and D, and Table S2). Considering the potential differentiation between IBs and tendons, we identified three tendon-related cell clusters based on the high expression of tendon markers (*tnmd* and *col1a1a*). We further defined the tendon progenitors based on the specific expression of *scxa*, which promotes early tendon development. In contrast, mature tenocytes were defined based on a high expression of *mkxa*, and the remaining cells, called differentiating tenocytes, were in a transitional state of differentiation from tendon progenitors to mature tenocytes according to the expression distribution of *tnmd*, *col1a1a* and *scxa* [16] (Fig. 1C and D). More cell markers were identified, such as *omd* and *aspn*, specifically expressed by mature tendons and osteoblasts, respectively (Table S2). Other cell populations were also annotated with epidermal, immune and skeletal muscle-specific gene signatures (Fig. 1 and Table S2).

### Delineating the osteoblast differentiation lineage and screening key genes regulating IB development

To understand the origin of IB-related osteoblasts, we delineated the osteoblast differentiation lineage during IB development using a trajectory model (monocle2) [17]. The pseudo-timing graphs and

**Figure 2.**
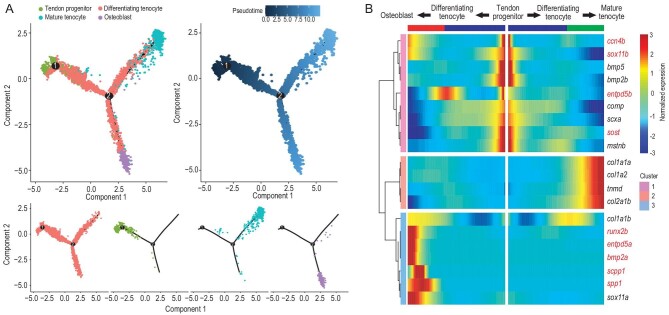
Differentiation trajectory construction of tendons and intermuscular bones, and screening of key regulators for IB formation. (A) Diffusion map visualization of the tendon and IB trajectories simulated by monocle2 across tendon progenitors, mature tenocytes, differentiating tenocytes and osteoblasts on the left. The corresponding diffusion pseudo-time is indicated in the bottom-left frame. A diffusion map visualization of each cluster is shown on the right. (B) Heatmap of the gene expression (smoothed over three adjacent cells) in subsets ordered by the pseudo-time of mature tenocytes and osteoblasts, as in (A); some curated genes are shown.

 

diffusion maps allowed us to reconstruct the differentiation trajectory of tendon progenitors into mature tenocytes or osteoblasts (Fig. 2A). Next, we performed functional annotation for the marker genes of the four cell clusters to explore their biological functions. Gene ontology (GO) analysis showed that osteoblast cluster genes were enriched in the following two terms: regulating anatomical structure development and multicellular organism development (Fig. S3A). By comparing the expression patterns of these genes, we identified genes that play a potential regulatory role in specific cell types, such as *col12a1b* and *col27a1b*, which were highly expressed in osteoblasts (Fig. S3B). We also revealed mineralization-related genes that were highly expressed in osteoblasts (*entpd5a*, *sox11*, etc.; Fig. S3C) [18,19], as well as osteogenesis and tendon development regulatory genes revealed by earlier studies [8,13,18–22]. KEGG analysis showed that osteoblast cluster genes (*id2a*, *thbs1b*, *nog3*, *nog2*, *tgfb3*) were enriched in TGF-β/BMP pathway signaling (Fig. S4D and Table S2). To further address the molecular mechanisms of IB development, we used more strategies to screen key genes for IB formation. Previous studies have screened *ccn4b* in relation to IB formation in tambaqui by comparing natural mutant individuals (without IBs) and wild individuals (with IBs) [9]. Some genes involved in bone development (*runx2, bmp2*, *enptd5*, etc.) and tendon development (*tnmd*, *col1a1*, *col2a1*, etc.) were also selected as the key candidate genes involved in IB formation. Through analyzing their expression patterns in the differentiation trajectory from tendon progenitors to mature tenocytes or osteoblasts, we identified 10 key candidate regulatory genes (*runx2a, runx2b, sost, bmp2a, scpp1, spp1, entpd5a, entpd5b, sox11b* and *ccn4b*) for IB formation because of their high expression in osteoblasts (Fig. 2B).

### Loss of *runx2b* completely inhibits IB formation

**Figure 3.**
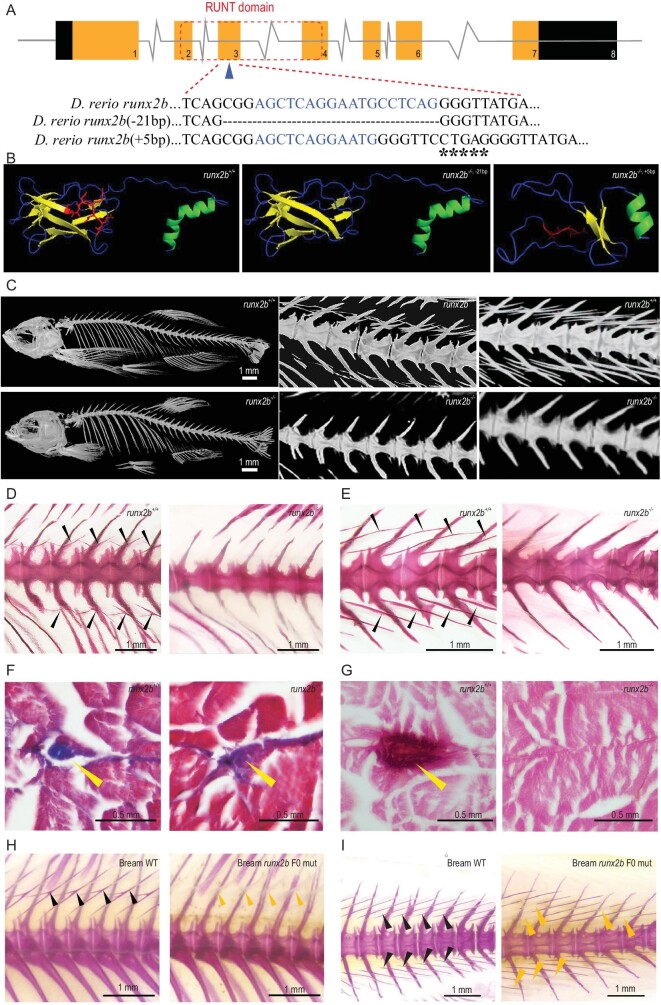
Gene structure and phenotypic characteristics of *runx2b*^+/+^ and *runx2b^−/−^*fish. (A) *runx2b* gene structure and RUNT domain characteristics. CRISPR-Cas9 target sequences are marked as blue letters in the DNA sequences. Deleted nucleotides are shown by ‘-’ and inserted sequences are marked as ‘*’ in *runx2b*^−/−^ zebrafish. (B) The runx2b protein structure. The β-sheet domain changed in *runx2b^−/−^* zebrafish. The red area indicates the missing or changed amino acids in *runx2b^−/−^* zebrafish compared with those in *runx2b*^+/+^ zebrafish. The α-helix is indicated in green; β-sheet is indicated in yellow; random coil is indicated in blue. (C) Micro-CT scan with a resolution under 6 μm showing overall skeletal structures. Regions of vertebrae 9–14 and 19–23 were scanned with micro-CT under 4 μm resolution to show details of the IBs. (D and E) Alizarin red S staining of the abdominal and caudal region. IBs (arrowheads) are lost in *runx2b^−/−^* zebrafish. (F) Masson's trichrome staining of transverse paraffin sections. The general tissue organization is maintained in *runx2b^−/−^* zebrafish compared to that in *runx2b*^+/+^ zebrafish. (G) Transverse paraffin sections of the area normally containing IBs (arrowheads) stained with Alizarin red S, indicated the loss of mineralized IBs in *runx2b^−/−^* zebrafish. (H and I) IBs phenotype of *runx2b* F_0_ generation mutants in blunt snout bream. The IBs are partially lost in the tail and back parts of the *runx2b* F_0_ generation mutants. The black arrowhead indicates mineralized IBs, while yellow arrowhead indicates incomplete mineralized IBs.

To further determine the key genes regulating IB formation, we constructed zebrafish mutant lines for 10 candidate genes with the CRISPR-Cas9 method (Table S3) to assess their function. Bone staining showed that *entpd5a^−/−^* (40 dpf) and *runx2b^−/−^* mutants totally lacked IBs, whereas the other eight mutants had normal IB phenotypes at 90 dpf (Fig. S4 and Table S4). Intriguingly, unlike *entpd5a^−/−^* mutants, which died at ∼40 dpf, the *runx2b^−/−^* mutants survived normally. Additionally, five-month-old *runx2* double mutants (*runx2a^−/−^/runx2b^−/−^*) not only completely lacked IBs, but their vertebrae also showed severe curvature (Fig. S5). The phylogenetic and structural analysis of *runx2* in different species showed that *runx2b* in fish with IBs clustered independently from *runx2* in fish without IBs, while the AD_2_ (Activating domain 2) showed obvious differences between fish with and without IBs (Fig. S6). Gene structure analysis showed that either a 21 bp deletion or a 5 bp insertion in the 3rd exon of *runx2b* resulted in significantly deviating *runx2b* protein and IB phenotypes compared to that in the wild type (Fig. 3A and B). Micro computed tomography (Micro-CT) and bone staining showed that the IB was completely lost in mutants (Fig. 3C–E, Figs S7 and S8). Alizarin red S and Masson's trichrome staining of transverse paraffin sections also confirmed that calcified tissue was absent from the tendons of *runx2b^−/−^* compared with *runx2b*^+/+^ zebrafish (Fig. 3F and G). The two mutant lines (a 21 bp deletion and a 5 bp insertion in the 3rd exon of *runx2b*) possessed the same phenotype (completely lost IB), showing that the phenotypes were indeed caused by loss of *runx2b*.

Next, we successfully used the CRISPR-Cas9 method to construct the F_0_ mutant of *runx2b* by targeting three different sites in the farmed fish *M. amblycephala* (Table S3). The mutation rate of each target in 902 individuals was 21.95%, 25.94% and 18.07%, respectively (Table S4-2). We randomly selected 54 F_0_ mutant fish at 70 dpf and Alizarin red S staining showed obvious IB phenotype in the 6 F_0_ mutants (11.1%) (Fig. 3H–I), with the IB number being reduced by 5.6%–33.8% (Table S4-3). As it takes two years for *M. amblycephala* to mature, another four years will be needed to obtain homozygous mutations of *runx2b* in this species. Nevertheless, the obvious IB phenotype in the F_0_ generation strongly suggests that *runx2b* plays a key role in regulating the IB formation of fish.

### Loss of *runx2b* produces no obvious differences in development and growth of other bones, swimming performance or muscle nutrient content

**Figure 4.**
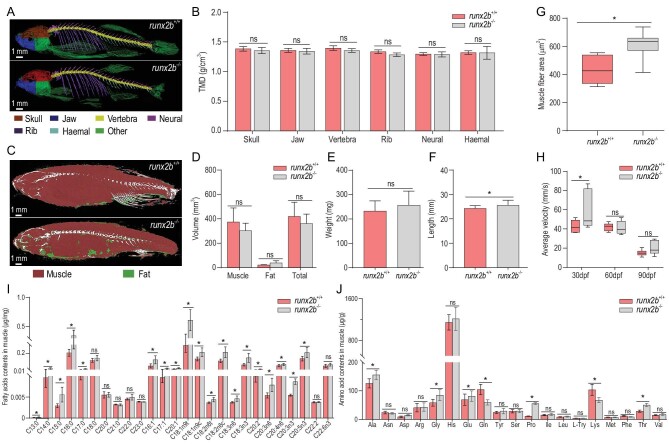
Comparison of characteristics of other bones, swimming performance and muscle nutrient content between *runx2b*^+/+^ and *runx2b*^−/−^ zebrafish. (A and B) Tissue mineral density (TMD) value of six distinctive bone elements. (C and D) Quantification of muscle and fat volumes. (E and F) Body weight and body length. (G) Muscle fiber area of tail muscles, calculated based on hematoxylin and eosin (HE)-stained sections. (H) Average swimming velocity. (I) Fatty acid contents in the muscle tissue of zebrafish at 90 dpf. (J) Amino acid contents in the muscle tissue of zebrafish at 90 dpf. ns, *P* > 0.05; *, *P* < 0.05.

Micro-CT analysis showed no differences in tissue mineral density (TMD) between *runx2b^−/−^* and *runx2b*^+/+^ zebrafish (Fig. 4A and B) for vertebrae, ribs, haemal spines and neural spines (*P* > 0.05), indicating that the loss of *runx2b* did not strongly affect the mineralization of bones other than IBs. Likewise, Alizarin red S staining and *in vivo* observations using green fluorescence showed that there were no obvious differences in the skull between *runx2b^−/−^* and *runx2b*^+/+^ zebrafish (Fig. S9A and B). In addition, no significant differences in the pattern and number of pharyngeal teeth could be observed between *runx2b^−/−^* and *runx2b*^+/+^ zebrafish (Fig. S9C and D).

Total muscle volume and body weight showed no obvious differences between the *runx2b^−/−^* and *runx2b*^+/+^ zebrafish at 90 dpf (*P* > 0.05; Fig. 4C–E), whereas the body length of *runx2b^−/−^* zebrafish was 5% greater than that of *runx2b*^+/+^ zebrafish (*P* < 0.05; Fig. 4F). The cross-sectional area of the muscle fibers in mutant fish was larger (*P* < 0.05; Fig. 4G). The average swimming velocity in *runx2b^−/−^* zebrafish at 30 dpf was significantly higher than that of *runx2b*^+/+^ zebrafish, but without significant differences at 60 dpf and 90 dpf (Fig. 4H). Thus, our results showed that *runx2b* deletion has no obvious adverse effects on zebrafish growth and swimming performance.

To assess the nutrient content of the *runx2b^−/−^* and *runx2b*^+/+^ zebrafish, the amino acid (AA) and fatty acid (FA) contents between *runx2b^−/−^* and *runx2b*^+/+^ zebrafish were compared at 90 dpf, respectively. *runx2b^−/−^* mutants had more abundant monounsaturated FAs (MUFAs) and polyunsaturated FAs (PUFAs), such as eicosapentaenoic acid (EPA) (20:5n3) and oleic acid (18:1n9), than *runx2b*^+/+^ zebrafish (Fig. 4I). For most of the identified AA content, the results showed no significant differences between *runx2b^−/−^* and *runx2b*^+/+^ zebrafish (Fig. 4J).

### Osteoblasts are nearly absent in *runx2b^−/−^* mutants and the TGF-**β**/BMP signaling pathway plays an important role in IB formation

**Figure 5.**
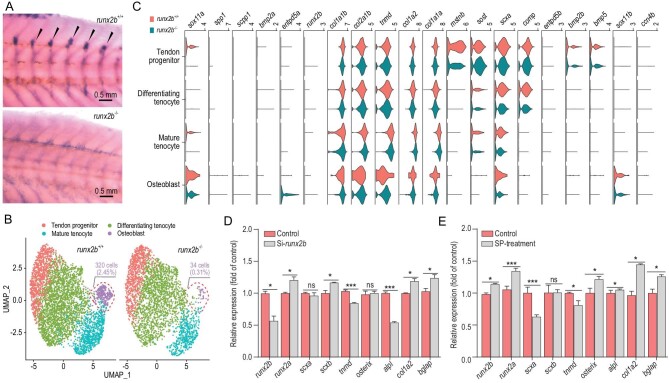
Comparative transcriptome analysis of *runx2b*^+/+^ and *runx2b^−/−^* zebrafish, and overexpression and knockdown analysis of *runx2b*. (A) *In situ* hybridization of *runx2b* in the tissue of origin of IBs (IBs, shown with arrowheads). The expression of *runx2b* was significantly reduced in *runx2b^−/−^* zebrafish. (B) UMAP visualization of tendon progenitors, mature tenocytes, differentiating tenocytes and osteoblasts in *runx2b*^+/+^ and *runx2b^−/−^*zebrafish. The ratios of osteoblasts to the total number of cells in *runx2b*^+/+^ and *runx2b^−/−^* zebrafish were 2.44% (340 cells) and 0.31% (34 cells), respectively. (C) Violin plots showing expression of differentially expressed genes in each cluster related to IB formation between *runx2b*^+/+^ and *runx2b^−/−^*zebrafish. (D, E) Overexpression and knockdown analysis of *runx2b* in IB cells of *M. amblycephala*. The expression levels of genes related to osteoblasts were detected by qRT-PCR. ANOVA (analysis of variance) was used to test the differences in expression. ns, *P* > 0.05; *, *P* < 0.05; **, *P* < 0.01; ***, *P* < 0.001.

The absence of IBs caused by the decreased transcription and translation levels of *runx2b* (Fig. 5A and Fig. S10A) is evidently accompanied by changes in the expression of interacting regulatory factors. To identify the gene expression profile after the loss of *runx2b*, a bulk RNA sequencing analysis of the tissue normally containing the IBs of *runx2b^+/+^* and *runx2b^−/−^* zebrafish was conducted. We identified 655 differentially expressed genes, including 395 downregulated and 260 upregulated genes (Fig. S10B). Functional annotation further revealed the key genes that were significantly enriched in osteogenic differentiation pathways (TGF-β/BMP pathway, mitogen-activated protein kinase (MAPK) signaling pathway, etc.; Table S5). Through quantitative real-time PCR (qRT-PCR) analysis, we verified the changes in expression of these genes in relation to tendon development, osteoblast differentiation and mineralization, such as *osterix*, *alpl*, *entpd5a* and *bglap*, after *runx2b* deletion (Fig. S11). In this way, we collected basic molecular information to clarify the regulatory mechanism of IB development.

To further investigate the effect of the absence of *runx2b* on IB-related cell differentiation and proliferation, we performed scRNA-seq on the tissue of origin of IBs in 60 dpf *runx2b^−/−^* and *runx2b*^+/+^ zebrafish (Fig. S12). In the mutants, osteoblasts had almost disappeared, and the percentages of tenocyte subpopulations compared to the total cell number also showed varying degrees of decline in *runx2b^−/−^* fish (Fig. 5B). Moreover, by comparing gene expression between the *runx2b*^+/+^ and *runx2b^−/−^* corresponding cell clusters (Table S6), we found that apoptosis-associated genes (*fsta*, *fos*, *jun*, *fosab*), a cell growth inhibitor (*cgref1*) and inflammation-related genes (*fstl1b*, *il4r.1*) were upregulated, whereas genes related to bone mineralization (*bmp2a*, *bmp2b*, *scpp1*, *spp1*, *col1a2*, etc.) were downregulated in mutants (Fig. 5C). This could explain the significant decrease in the number of osteoblasts in *runx2b^−/−^* fish [23–25]. Moreover, the functional annotation results showed that the differentially expressed genes between *runx2b*^+/+^ and *runx2b^−/−^* osteoblast clusters were significantly enriched in the TGF-β/BMP pathway (Fig. S13), including the upregulated genes *id1*, *id3*, *pitx2* and *bmp5* and the downregulated genes *nog2*, *id2a* and *thbs1b* (Table S6), again revealing the key role of the TGF-β/BMP pathway in IB development. In addition, we conducted RNA interference and expression induction based on *runx2b* at the cellular level and found that the expression of tendon and osteoblast marker genes was consistent with the expression changes of *runx2b*. For example, expression of the osteoblast transcription marker *alpl* was significantly downregulated upon *runx2b* RNA interference and upregulated in the SP (neuropeptide substance P) induction experiment at the cellular level (Fig. 5D and E and Fig. S14 and 15). This clarified the promoting effect of *runx2b* on osteoblast differentiation and IB formation.

## DISCUSSION

IBs, which are present in the muscle tissue of many important farmed fish species, can be dangerous for consumers as they can injure the oropharynx [6–8]. Previously, a strategy has been devised to mitigate the problem by breeding normal IB-free individuals with advanced breeding technology. This strategy became more promising with the accidental discovery of individuals in a tambaqui breeding population that had no IBs [9]. For a controlled breeding of cyprinid fish species without IBs, significant progress could be made by understanding the mechanism of IB formation [8,13,26] and by identifying the key genes that regulate the process. Recent studies have demonstrated the involvement of tendons and osteoblasts in IB formation [8,27]. Here, we specifically identified four cell clusters related to tendons and IBs (tendon progenitor cells, mature tenocytes, differentiating tenocytes and osteoblasts). We constructed the pseudo-differentiated trajectory, showing that IBs originate from the root state (tendon progenitors) to the differentiating tenocyte, and finally differentiate into osteoblasts, forming IB. This differentiated trajectory not only provided indirect evidence for the hypothesis that the IBs derive from tendons, but also provided the theoretical basis for the study of the molecular mechanism of IB differentiation.

According to our scRNA-seq analysis, we identified tendon progenitors, differentiating tenocytes, mature tenocytes and osteoblasts related to IB formation based on marker gene expression. *scx* is a basic helix-loop-helix transcription factor that is predominantly expressed in the tendon/ligament cell lineage. *scx* is the earliest known marker for tendon progenitor cells and positively regulates the expression of *tnmd* and *col1a* in tenocytes [28,29]. *entpd5* serves as a marker for osteoblasts; it is expressed in osterix-expressing cells and tissues associated with skeletal mineralization [18]. TNC (Tenascin C) is an extracellular matrix glycoprotein involved in osteogenesis and bone mineralization. Inhibiting *tnc* expression can decrease early marker gene expression for osteoblast differentiation (*col1a1* and *alp*) [30]. Here, we provide evidence that *entpd5a* and *tnc* have a higher expression in osteoblasts, further supporting that the osteoblasts are involved in IB formation. Subsequently, a pseudo-time atlas of the four cell groups revealed the differentiation trajectory of tendon progenitors into mature tenocytes or osteoblasts (Fig. 2). Intriguingly, we found that a mesenchymal stem cell (MSC) group with osteogenic potential did not exist in the tissue containing IBs, indicating that IBs might not derive from MSCs but from a tendon-related cell subgroup. This result suggests that IBs originate from tendon cells that differentiate into osteoblasts.

Identification of the core cell differentiation trajectory of IB formation provided us with important information to further screen specific regulatory genes. Mutants of 10 candidate genes were constructed with the CRISPR-Cas9 gene editing method in zebrafish. We successfully obtained *runx2b*^–/–^ mutant zebrafish strains without IBs and with stable inheritance. *runx2* is a well-known transcription factor and has a crucial role in osteoblast differentiation. It can bind *osterix, alpl* and *col1a1* promoter sites as well as regulate the expression of bone matrix genes (*bglapl*, *spp1*) [21,22,31]. As a result of genome duplication in teleosts, zebrafish have two orthologs of the mammalian *runx2* gene, *runx2a* and *runx2b* (19% difference at the amino acid level), with similar but not identical expression patterns during development [32]. The present study showed the differences in IB phenotype between *runx2a* (with IBs) and *runx2b* (without IBs) zebrafish stable mutants. Unlike *Runx2* mutant mice, which are dead at the embryonic stage [33], both *runx2a* and *runx2b* zebrafish mutants are viable, fertile and display normal mineralization in their skeleton, except for the IB defect in *runx2b^−/−^* zebrafish. This strongly suggests that *runx2b* is more significant for IB formation or mineralization than *runx2a*. Our expression analysis showed that *alpl*, *osterix*, *bglapl* and *spp1* were significantly downregulated in *runx2b^−/−^* zebrafish, indicating that loss of *runx2b* in zebrafish can inhibit the normal expression of osteogenic genes, thereby limiting the formation and mineralization of IBs.

The TGF-β/BMP signaling pathway plays an important role in the regulation of osteoblast lineage-specific differentiation and later bone formation [13,34], and interacts with various factors and pathways, including transcription factor *runx2* [34]. Previous studies have demonstrated that *scx* and *osterix* are mutually regulated by the activation of BMP signaling [29]. *Scxa* mRNA was detected in various skeletal elements of zebrafish, such as the intermuscular tendons at the vertical myosepta, the fin radials and the joints in the fin rays [15]. Our previous study showed that a loss of *scxa* in zebrafish results in a defect of IBs and ribs and negatively regulates genes of BMP signaling (*bmp2a, bmp2b*, etc.) [8]. Comparative transcriptome analysis at the tissue and single-cell levels in this study showed that many differentially expressed genes were significantly enriched in the TGF-β/BMP pathway, including *bmp5*, *id1*, *id2a*, *id3* and *nog2*, showing that TGF-β/BMP signaling might be involved in IB formation. However, this work represents only a preliminary attempt to reveal the significance of TGF-β/BMP signaling for IB formation based on scRNA-seq. The actual regulatory relationships may be more complex and thus, further validation assays will be needed for revealing how TGF-β/BMP signaling regulates IB formation.

Although the decisive role of *runx2b* in IB formation was only confirmed in the model zebrafish in this study, the similarity of development of IBs in zebrafish compared to other aquaculture species, especially carps, suggests the practical feasibility of breeding IB-free aquaculture species through knocking out *runx2b*. Zebrafish and carp species have the same IB types, epineurals and epipleurals, which are all ossified from anterior to posterior from ∼20 dph (days post-hatching) to 40 dph [35]. Moreover, the complete absence of IBs has been identified in tambaqui individuals from one breeding population in Brazil, as well as in one gynogenetic grass carp (*C.idella*) in China [36]. This suggests that the IB phenotype may be genetically controlled by one recessive mutant allele due to homozygosity engendered by gynogenesis, or by other, possibly environmental, factors. Our attempts at generating IB-free individuals of the economically important aquaculture species *M. amblycephala* have not reached the F_2_ generation with *runx2b^–^^/^^–^* homozygous individuals. Yet, the F_0_ population resulting from *runx2b* gene editing already showed a significant decrease in IB number. These results confirm the key role of *runx2b* in IB formation. Nevertheless, the complexity of regulatory mechanisms in different teleost fish taxa may lead to limitations in the application of our results. The skeletal development, growth, swimming performance and muscle nutrient content in the aquaculture species also need to be thoroughly evaluated once IB-free strains are obtained.

## CONCLUSION

This study reveals that teleost IBs originate from tendons in the skeletal muscle and that the differentiation trajectory from tendon progenitors to osteoblast lineage is key for IB formation. Large-scale gene function analysis using CRISPR-Cas9 identified the crucial role of *runx2b* in IB formation. Loss of *runx2b* significantly reduced osteoblast differentiation and inhibited IB formation, potentially providing a basis for breeding strains of farmed fish without IBs. This would improve their safe consumption. Our results also provide data and directions for further studies. In particular, clarifying the specific regulatory mechanism of TGF-β/BMP signaling on IB formation could possibly provide additional tools for a targeted prevention of IB development.

## MATERIALS AND METHODS

A detailed description of the materials and methods is available in the supplementary data.

## DATA AVAILABILITY

The raw sequencing data of the scRNA-seq and bulk-seq were submitted to National Center for Biotechnology Information (NCBI) under the accession number PRJNA733247. All other supporting data are available in the supplementary data.

## Supplementary Material

nwac152_Supplemental_Files
